# Outbreak of SARS-CoV-2 Lineage 20I/501Y.V1 in a Nursing Home Underlines the Crucial Role of Vaccination in Both Residents and Staff

**DOI:** 10.3390/vaccines9060591

**Published:** 2021-06-02

**Authors:** Andrea Orsi, Alexander Domnich, Vanessa De Pace, Valentina Ricucci, Patrizia Caligiuri, Livio Bottiglieri, Rosanna Vagge, Maurizio A. Cavalleri, Francesco Orlandini, Bianca Bruzzone, Giancarlo Icardi

**Affiliations:** 1 Department of Health Sciences (DISSAL), University of Genoa, 16132 Genoa, Italy; andrea.orsi@unige.it (A.O.); icardi@unige.it (G.I.); 2 Hygiene Unit, San Martino Policlinico Hospital-IRCCS for Oncology and Neurosciences, 16132 Genoa, Italy; Vanessa.DePace@hsanmartino.it (V.D.P.); valentina.ricucci@hsanmartino.it (V.R.); patrizia.caligiuri@hsanmartino.it (P.C.); bianca.bruzzone@hsanmartino.it (B.B.); 3 Laboratory Medicine, San Martino Policlinico Hospital-IRCCS for Oncology and Neurosciences, 16132 Genoa, Italy; livio.bottiglieri@hsanmartino.it; 4 Local Health Unit 4, 16043 Chiavari, Italy; rosanna_vagge@libero.it (R.V.); maurizioalessandro.cavalleri@asl4.liguria.it (M.A.C.); francesco.orlandini@asl4.liguria.it (F.O.)

**Keywords:** SARS-CoV-2, COVID-19, vaccination, nursing home, elderly, Italy

## Abstract

Elderly residents in nursing homes are at very high risk of life-threatening COVID-19-related outcomes. In this report, an epidemiological and serological investigation of a SARS-CoV-2 outbreak in an Italian nursing home is described. Among the residents, all but one (19/20) were regularly vaccinated against SARS-CoV-2. In mid-February 2021, a non-vaccinated staff member of the nursing home was diagnosed with the SARS-CoV-2 infection. Following the outbreak investigation, a total of 70% (14/20) of residents aged 77–100 years were found positive. The phylogenetic analysis showed that the outbreak was caused by the SARS-CoV-2 variant of concern 202012/01 (the so-called “UK variant”). However, all but one positive subjects (13/14) were fully asymptomatic. The only symptomatic patient was a vaccinated 86-year-old female with a highly compromised health background and deceased approximately two weeks later. The subsequent serological investigation showed that the deceased patient was the only vaccinated subject that did not develop the anti-spike protein antibody response, therefore being likely a vaccine non-responder. Although the available mRNA SARS-CoV-2 vaccine was not able to prevent several asymptomatic infections, it was able to avert most symptomatic disease cases caused by the SARS-CoV-2 variant of concern 202012/01 in nursing home residents.

## 1. Introduction

Residents of long-term care facilities (LTCFs) are at disproportionately high risk of severe outcomes following the SARS-CoV-2 infection [1,2]. The overwhelming majority of LCTF residents is represented by older adults and the so-called “oldest old” individuals aged 80 years and above [3]. The available systematic research [4,5,6] affirms that older age per se is among the principal risk factors of both severe disease progression and mortality. On the other hand, elderly residents of LTCFs are systematically different from community-dwelling older adults. For instance, almost all of them are affected by severe co-morbidities [7,8]. In their meta-regression analysis, Patel et al. [9] documented that after adjustment for age, comorbidities such as previous cerebrovascular or cardiovascular diseases were still significant independent predictors of poor COVID-19-related outcomes. This may further worsen the course of disease in the institutionalized elderly. In this regard, it has been estimated [10] that, during the first pandemic wave, the mortality rate among the Italian elderly residents in LTCFs was 8.5%.

Unlike the community-dwelling elderly, LCTF residents live in close proximity with caregivers moving throughout the building and, therefore, the risk of infection spread is increased [11]. Moreover, contractors and workers of one LCTF may also be employed in another LCTF, that may further facilitate the spread of SARS-CoV-2 [12].

A rapid diffusion of the SARS-CoV-2 lineage 20I/501Y.V1 (variant of concern 202012/01, the so-called “UK variant”) across Europe has raised issues on the possibility of the increased disease infectiousness and severity [13] and lower effectiveness of the authorized vaccines [14]. Thus, Davies et al. [15] have reported that the novel variant has a 43–90% higher reproduction number than the previously circulating strains. In Italy, according to GISAID EpiCoV [16] the first documented cases of the SARS-CoV-2 lineage 20I/501Y.V1 were detected in mid-December 2020; since that time, the novel variant dominated the epidemiological scene.

According to the Italian guidelines [17], both residents and staff of LTCFs are the top priority groups for SARS-CoV-2 vaccination; the messenger RNA (mRNA) vaccine BNT162b2 (Comirnaty, Pfizer-BioNTech) was mostly used during the campaign started in late December 2020. The available phase III randomized controlled trial [18] suggests a high efficacy (>94%) of the BNT162b2 vaccine in the elderly. The first real-world effectiveness data [19,20] are in line with these estimates. However, residents of LTCFs were usually excluded from these studies. A preliminary temporal (from 5th October 2020 to 14th March 2021) analysis conducted by researchers of the National Institute of Health [21] showed a substantial reduction in the number of novel infections, hospitalizations and deaths in a sample of Italian LTCFs from before to after the start of vaccination campaign.

In this paper, we report results of the investigation of an outbreak caused by the novel SARS-CoV-2 variant in an Italian LCTF that occurred after the start of the national immunization campaign. A particular attention will be paid to serology findings.

## 2. Description of the Nursing Home

The LTCF investigated is a 25-bed nursing home located in the Metropolitan City of Genoa (Liguria, northwest Italy). Liguria is among the “oldest” regions in Europe: a total of 28.7% of the population ages 65 years and above [22]. The LTCF staff is composed of 15 people: eight unlicensed assistive personnel members (UAPs), two nurses, two administrative workers (both in smart working during the investigation period), a visiting physiotherapist, an animator and a janitor. Both single and double occupancy rooms are available in the building and several common spaces (dining room, physiotherapy gym, gardens) are accessible to the residents.

During the investigation period, a total of 20 elderly (75% females) aged 77–100 years were living in the LTCF. Most (60%, 12/20) residents sojourned in double rooms. All staff members and residents were regularly screened for the SARS-CoV-2 infection by using an antigen-detecting rapid diagnostic test (Ag-RDT). The LTCF was closed for visitors from the beginning of the second pandemic wave.

## 3. Vaccination Campaign

In January 2021, all residents and staff members were offered a free-of-charge vaccination with the mRNA vaccine BNT162b2. In particular, all but one (19/20) resident were regularly administered two doses (on 14th January and 5th February 2021) of the vaccine. The remaining resident refused vaccination. By contrast, only 3 of 15 (both nurses and an UAP) staff members were regularly vaccinated. No serious adverse events following immunization occurred.

## 4. Description of the Outbreak

On 18th February, a previously asymptomatic, non-vaccinated UAP asked for sick leave and her nasopharyngeal specimen swabbed the day before resulted positive on the real-time reverse transcription polymerase chain reaction (RT-PCR). On this notice, an outbreak mitigation plan was implemented by the LTCF staff according to the available national guidelines [23]. On 19th February, all the residents underwent the Ag-RDT and 45% (9/20) resulted positive. On 20th February, all residents and staff were tested in a one-step multiplex RT-PCR on Biorad CFX96™ thermal cycler (Bio-Rad Laboratories, US) and a total of 12 residents and other three UAPs turned out positive. On the second RT-PCR testing performed a week later (on 26th February) other two positive results were documented. In sum, a total of 14 (70%) positive cases were identified.

The positivity rate among the residents living in double occupancy rooms (92%, 11/12) was significantly (Fisher exact test: *p* = 0.018) higher than those living in single rooms (38%, 5/8). Out o 14 positive residents, all but one were regularly vaccinated. From the clinical point of view, 13 positive patients were asymptomatic for any influenza-like illness (ILI) or acute respiratory infection. The only symptomatic resident was a vaccinated 86-year-old female who developed some ILI symptoms, including moderate grade fever, dry cough and malaise. From her history, the patient had long-term sequelae of ischemic stroke (difficulties in functional ambulation), a recently surged epilepsy and severe chronic venous insufficiency. The patient was hospitalized on 24th February and deceased on 3rd March (Table 1).

**Figure 1:**
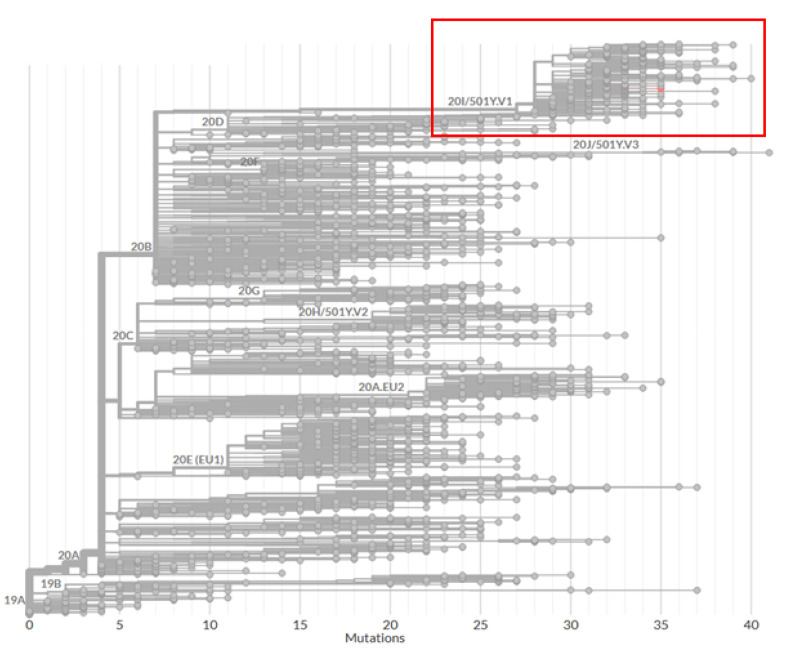
Phylogenetic tree analysis of the full-genome sequence of the strain detected in the deceased resident (analysis was performed using the online application Nextclade beta [24]).

The SARS-CoV-2 RNA detected from the only symptomatic patient’s swab was then sequenced (Illumina MySeq by Illumina, US) and identified as lineage 20I/501Y.V1 (Figure 1) [24]. A random sample of five asymptomatic positive swabs collected on 20th February were also sequenced and the identical genetic sequence was established.

All but two residents negativized by 10th March. On 15th March, the remaining two positive residents turned out negative.

## 5. Serology Findings

On 22nd February, venous blood samples were taken from all 20 residents and analyzed for anti-N IgG and IgM (iFlash 1800 by YHLO, China) and anti-S1/S2 IgG neutralizing antibody responses (Liaison XL by DiaSorin, Italy). All 20 samples were negative on IgG/IgM anti-N response, suggesting that the residents were likely previously naïve to SARS-CoV-2. By contrast, 90% (18/20) of residents were positive on anti-S1/S2 neutralizing antibody response, suggesting that the S protein-based mRNA vaccine induced some IgG response. The two negative results belonged to the above-described non-vaccinated and deceased residents, respectively, suggesting that the latter was likely a vaccine non-responder. Among the vaccinated residents, those RT-PCR negative had at least (since the IgG concentration of the specimens was right-censored at 400 AU/mL) 2.3 times higher geometric mean concentration (GMC) than RT-PCR positive patients (292 vs. 124 AU/mL). No significant (*p* = 0.15) correlation between anti-S1/S2 IgG level and age was found (Spearman’s ρ = −0.34).

**Figure 2:**
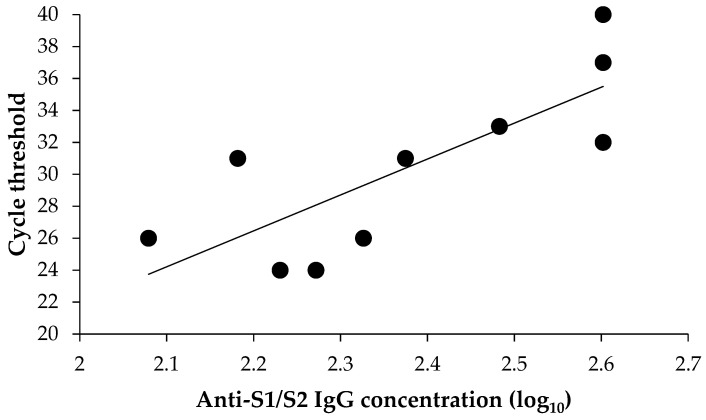
Association between anti-S1/S2 IgG concentration and viral load.

Among positive vaccinated individuals, 11 had detectable anti-S1/S2 antibodies. One sample was associated RNA-dependent RNA polymerase (RdRp)/S gene target failure and was excluded from the correlation analysis. As shown in Figure 2, there was a clear linear association (R^2^ = 61.7%) between the anti-S1/S2 IgG level and viral load proxied as RT-PCR cycle threshold parameter for the RdRp/S gene region (lower cycle threshold values indicate higher viral loads). In particular, higher anti-S1/S2 IgG concentrations were associated (*p* = 0.007) with higher cycle threshold values (Figure 2).

The second blood collection was performed on 15th March. All alive (*n* = 19) residents were positive on anti-S1/S2 IgG with a GMC of 252 AU/mL, that was not different (Wilcoxon test: *p* = 0.43) from that observed on 22nd February (GMC: 163 AU/mL). A total of ten (53%, 10/19) developed detectable anti-N IgG levels and all of them were positive on RT-PCR. By contrast, all negative subjects did not mount a detectable anti-N IgG response. No correlation (Spearman’s ρ = 0) between anti-N and S1/S2 IgGs was found.

## 6. Discussion

This outbreak investigation supports the current guidelines on SARS-CoV-2 vaccination [17] to prioritize healthcare workers, LTCF residents and other extremely vulnerable population groups. If not previously vaccinated, newly arrived residents and newly hired employees should be offered vaccination immediately. Indeed, considering that the LCTF was closed for visitors, the SARS-CoV-2 lineage 20I/501Y.V1 infection was most likely spread by a non-vaccinated UAP during her asymptomatic incubation period. On the other hand, even though most (13/19) vaccinated residents were infected, all but one were fully asymptomatic for the SARS-CoV-2 infection, while the only non-vaccinated subject became positive with the highest viral load (cycle thresholds for E, RdRp/S and N genes of 22, 25 and 21, respectively). This suggests that the BNT162b2 vaccine was able to prevent most symptomatic cases caused by the “UK” variant. We will now discuss our main results by making parallels with the available literature.

Although most LCTF residents were vaccinated, the present report shares some features with the first familial cluster of the 20I/501Y.V1 variant occurred in the northeast of Italy [25]. In particular, in their case report, Lo Menzo et al. [25] have described a family cluster initiated by a fully asymptomatic individual returning from London. The secondary transmission occurred during a Christmas lunch that is traditionally long and guests are used to move around. The attack rate was extremely high and eight out of nine individuals got infected; out of the infected individuals, four had mild symptoms, while another four were hospitalized. Among the hospitalized subjects, a 70-year-old male with a history of cardiovascular disease and advanced chronic kidney disease deceased two days after hospital encounter. The only RT-PCR negative subject had high titers of SARS-CoV-2-specific antibodies, suggesting a previous infection.

Few data on the effectiveness of SARS-CoV-2 vaccines in LTCF residents are available up to date. In a Danish cohort study [26], the adjusted effectiveness against both symptomatic and asymptomatic cases in LTCF residents (*n* = 39,040; mean age 84 years) was 52% (95% CI: 27–69%) and 64% (95% CI: 14–84%) during the periods of 0–7 and >7 days after the second dose, respectively. Although the present report was not designed nor powered to estimate vaccine effectiveness, the vaccination failure rate against any (asymptomatic or symptomatic) RT-PCR-confirmed infection observed is consistent with the findings of the Danish study [26]. In a recent US study [27], the incidence of SARS-CoV-2 in vaccinated (*n* = 13,048) LTCF residents was 1.0% within 2 weeks after receipt of the second dose. By contrast, the incidence was 4.3% in non-vaccinated residents. Similarly to our report, most positive cases were asymptomatic.

Although most available experimental and observational evidence has shown a clear protective effect of SARS-CoV-2 vaccination against disease, there is an ongoing debate on whether it can also block virus transmission. The transmission-blocking effect is difficult to quantify since a reduction of new infections in a region is also driven by other factors, such as behavioral changes and lockdown measures [28]. This also makes it difficult to compare estimates coming from different areas. A recent retrospective cohort study by Tande et al. [29] has established a vaccine effectiveness of 80% (95% CI: 56–91%) against asymptomatic SARS-CoV-2 infection after two doses of the mRNA vaccines. An observational study from Israel [20] reported a higher estimate: in the period of ≥7 days after the second dose of BNT162b2 the vaccine effectiveness in the elderly was 88.5% (95% CI: 86.4–90.3%). Dagan et al. [19] have reported an effectiveness of the BNT162b2 vaccine against asymptomatic infection of 29% (95% CI: 17–39%), 52% (95% CI: 41–60%) and 90% (95% CI: 83–94%), during the periods of 14–20 days after the first dose, 21–27 days after the first dose and ≥7 days after the second dose, respectively. These studies are, however, affected by several limitations, including both a short follow-up period and limited number of swabs performed (and, therefore, some positive asymptomatic cases could have been missed) [28]. No data specific to the LTCF residents, which usually present a highly compromised health background, are available up to date.

The emerging evidence suggests that even if the available vaccines may not induce the sterilizing immunity, they are effective in reducing the viral load and disease severity. A preprint by Levine-Tiefenbrun et al. [30] has revealed that SARS-CoV-2 infections occurring 12−28 days after the first dose of the BNT162b2 vaccine had a four-fold reduction in viral loads, as compared with non-vaccinated RT-PCR positive subjects. Indeed, we documented a clear negative association between the vaccine-induced anti-S1/S2 IgG neutralizing antibody response and viral load. This may also explain a high proportion of asymptomatic patients among the vaccinated residents (95%, 18/19), which makes sense, since the BNT162b2 vaccine used in the LTCF targets the S protein. Therefore, establishment of an accurate correlate of protection would be extremely important [31].

The SARS-CoV-2 lineage 20I/501Y.V1 seems to be covered by the available vaccines. A recent Italian study by Rondinone et al. [32] has indicated a high level of cross-lineage reactivity between the previously circulating strains and 20I/501Y.V1 lineage. In particular, all tested samples from the previously infected patients (*n* = 12) developed neutralizing antibody titers of 1:160–1:320 against the novel 20I/501Y.V1 lineage and these latter were the same as those against the previously circulating variant. On the other hand, a two-fold decrease in neutralizing activity against the “UK variant” in serum samples obtained from subjects immunized with the BNT162b2 vaccine has been also reported [33]. In this study, we then observed a relatively low case-fatality rate of 7% (1/14). Previous European studies conducted in LTCFs during the pre-vaccination and “pre-variant” periods reported substantially higher estimates for the case–fatality rates. For instance, it was 20%, 27.6% and 29% in a Spanish [34], Irish [35] and French [36] LTCF, respectively.

More generally, this investigation underlines the importance of social distancing strategies to be adopted in LTCFs in case of a suspected SARS-CoV-2 infection; all but one resident living in a double occupancy room got infected, while this proportion was significantly lower among the elderly living in single rooms. Indeed, in a cohort study by Brown et al. [37], it has been shown that the COVID-19 incidence in LTCFs with high crowding index (defined as the number of residents per room and bathroom across an entire nursing home) was about twice (9.7% vs. 4.5%, *p* < 0.001).

Analogously, our results are in line with the European Centre for Disease Prevention and Control (ECDC) framework [38] on the use of Ag-RDTs in LTCFs; the routine use of Ag-RDTs for screening purposes is advisable for both outbreak investigations and contact tracing.

In conclusion, in this case report we showed that vaccination of LTCF residents was able to avert most symptomatic disease cases caused by the SARS-CoV-2 variant of concern 202012/01. We believe that this outbreak could have been avoided, if vaccination coverage among the LTCF staff had been higher. In Italy, hesitancy towards SARS-CoV-2 vaccines is increasing [39]. On 1st April 2021, an emergency decree on the compulsory SARS-CoV-2 vaccination for healthcare professionals was approved by the Italian government [40]. This decree can increase vaccine uptake, but there is also a risk of compromising trust between health professionals and their institutions [41]. The compulsory vaccination should be accompanied by other policy measures.

## Figures and Tables

**Table 1:** Characteristics of positive and negative residents.

ID	Age	Sex	Vaccine	Ag-RDT	RT-PCR					COVID-19 Symptoms	Hospital Encounter	Death
					20/02	26/02	04/03	10/03	15/03 ^1^			
1	93	F	+	−	+	−	−	−	NA	−	−	−
2	96	F	+	+	+	+	−	−	NA	−	−	−
3	95	M	+	+	+	+	+	+	-	−	−	−
4	86	F	+	+	+	NA ^2^	NA ^2^	NA ^2^	NA	+	+	+
5	77	F	−	−	+	+	+	−	NA	−	−	−
6	80	F	+	−	+	+	−	−	NA	−	−	−
7	91	F	+	+	+	+	+	−	NA	−	−	−
8	90	F	+	+	+	+	+	−	NA	−	−	−
9	86	F	+	+	+	+	−	−	NA	−	−	−
10	91	M	+	+	+	-	−	−	NA	−	−	−
11	83	M	+	+	+	+	−	−	NA	−	−	−
12	92	M	+	+	+	+	+	+	-	−	−	−
13	84	F	+	−	−	+	+	−	NA	−	−	−
14	100	F	+	−	−	+	−	−	NA	−	−	−
15	83	F	+	−	−	−	−	−	NA	−	−	−
16	84	M	+	−	−	−	−	−	NA	−	−	−
17	87	F	+	−	−	−	−	−	NA	−	−	−
18	86	F	+	−	−	−	−	−	NA	−	−	−
19	88	F	+	−	−	−	−	−	NA	−	−	−
20	93	F	+	−	−	−	−	−	NA	−	−	−

^1^ Only previously positive subjects were tested. ^2^ Patient deceased on 3 March 2021.

## Data Availability

All relevant data are within the article.
